# Reference Data on the Normal Abdominal Wall Anatomy and Baseline Characteristics in Seventy-One Nulliparous Women

**DOI:** 10.3389/jaws.2023.10940

**Published:** 2023-02-02

**Authors:** K. Woxnerud, G. Sandblom, C. Hedbeck, A. Olsson

**Affiliations:** ^1^ Hela Kvinnans Klinik, Stockholm, Sweden; ^2^ Stockholm Hernia Center, Stockholm, Sweden; ^3^ Department of Clinical Science and Education, Karolinska Institute, Stockholm, Sweden; ^4^ Department of Surgery, Södersjukhuset, Stockholm, Sweden; ^5^ Unilabs Radiology, Stockholm, Sweden

**Keywords:** pregnancy, diastasis recti abdominis, rectus diastasis, linea alba, postpartum

## Abstract

**Aims:** The aim of this study was to describe the prepartum anatomy of the abdominal wall in a cohort of nulliparous women, for use as a reference for management of patients with postpartum abdominal wall insufficiency with or without rectus diastasis.

**Materials and Methods:** Seventy-one women were examined with ultrasonography of the abdominal wall. The inter-recti distance (IRD), anatomical variations of the linea semilunaris, and the oblique muscles were assessed. The waistline was measured during activation and relaxation of the abdominal core. Participant characteristics were registered. Questionnaires regarding habitual physical activity (Baecke), low back pain (Oswestry), physical functioning (DRI), urinary incontinence (UDI-6 and IIQ-7), and quality-of-life (SF-36) were answered.

**Results:** Mean age was 30.5 years (range 19–50 years) and mean BMI 23.5 kg/m^2^ (range 18–37). Ultrasonography showed a mean IRD of 10 mm (range 3–24) at the superior border of the umbilicus, 9 mm (4–20) 3 cm above the umbilicus, and 2 mm (−5–10) 2 cm below the umbilicus. The mean thickness of the linea alba was 3 mm (1.5–5) and mean distances between the lateral edge of the rectus muscle and the external, internal, and transverse oblique muscles were 12 mm (−10–28), 1 mm (−14–13) and 15 mm (−14–32) at umbilicus level. Responses to the DRI, UDI-6, IIQ-7 and Oswestry questionnaires showed generally lower scores than the normal population whereas Baecke and SF-36 scores were similar.

**Conclusion:** This study provides baseline data on normal abdominal wall anatomy in a healthy nulliparous female cohort, as well as levels of activity, physical function, disability, and quality-of-life.

## Introduction

The abdominal wall supports stability and motion of the trunk and protects the abdominal viscera. The anatomy of the abdominal wall is affected by pregnancy, obesity, and other conditions that stretch and widen the muscles, fasciae, and skin. One important component of these abdominal wall deformations is widening of the linea alba, i.e., rectus diastasis (RD) (1).

The development of RD is a normal physiological anatomical change during pregnancy caused by mechanical stretching of the abdominal wall combined with hormonal changes (2). Approximately one-third of postpartum women are reported to have a persistent RD following pregnancy (3). RD has traditionally been defined as an inter-recti distance of 2.8–3 cm, based on a few studies (4, 5). In recent guidelines issued by the European Hernia Society (EHS), RD is defined as a separation of the rectus abdominis muscles exceeding 2 cm (6).

As research continues, our understanding of the structural and mechanical properties of the muscles and fasciae of the abdominal ventral wall has increased. Several studies describe the inter-recti distance (IRD) at different stages postpartum. Available research on nulliparous women have reported mean inter-recti distances in a range of 13–18 mm. Beer et al. assessed 150 nulliparous women with ultrasound in a supine position and measured the IRD at three different points (5). Balasch-Bernat et al. included 75 women, of whom 25 were nulliparous, in their measurement of the IRD (7). Tuominen et al. compared the IRD in nulliparous, with postpartum women (8). In Stecco’s measurement of the thickness of the abdominal muscles and fasciae, a total of 36 women were included of whom 13 were nulliparous (9, 10). The architecture of collagen fibres in the linea alba and rectus sheath is well described by Axer et al. in a study on 12 human cadavers (11).

Previous anatomical studies have thus focused on the linea alba, measuring the width and thickness of this fascial connection. The linea alba is well described in the literature but information is sparse on the normal anatomy and postpartum changes of muscles and fasciae in other regions of the abdominal wall such as the linea semilunaris. Linea semilunaris is the fascial connection between the medial aspect of the external oblique, the internal oblique and the transversus abdominis muscles connecting respectively to the lateral aspect of the rectus abdominis muscle. There are no reference values presented on this fascial connection in the literature although it may also be affected by the stretching forces during pregnancy.

All reported values of the linea alba, both clinically and during surgery, have been obtained in the supine position, which differ from most activities in everyday life, mainly performed in the upright standing or sitting position. From a biomechanical perspective, forces acting on the abdominal wall differ between these body positions. In the present study we therefore examine the RD in both supine lying as well as in standing to examine if the RD changes with body position in nulliparous women. We intend to define baseline values of the abdominal wall anatomy, to compare with measurements on postpartum women. To completely understand how the abdominal wall copes with RD, measures in relaxed supine position as well as during straining are needed.

The natural course from nulliparous state to the postpartum period is poorly described in previous studies and few studies have followed the natural course longitudinally. There is thus a need for studies with normative anatomic measures for nulliparous as well as multiparous women. Studies have shown an association between structural changes of the abdominal wall and both physical performance and functional disability (12). We need to understand postpartum anatomical changes in relation to functional demands, but there are no standardized data on normal physical function levels in the nulliparous population.

The main reasons for carrying out surgery for RD is, in addition to cosmetic improvement, to improve abdominal trunk function. More studies on the effectiveness of surgery for restoring continence and trunk function are needed. Such studies should be based on baseline data from women prior to surgery as well as nulliparous women in order to assess the actual benefit from the treatment. Ideally, randomized controlled trials may provide data on what RD surgery may accomplish in comparison with the outcome from conservative management. There is, however, a need for normative studies assessing the trunk function in nulliparous as well as multiparous women to fully understand how the symptoms change as the result of a pregnancy as well as from surgery aiming at restoring the function.

The aim of the present observational study was to increase our understanding of the normal abdominal wall anatomy in nulliparous women, before hormonal and structural changes appear during pregnancy. This study measures and systematically describes the normal anatomy of the abdominal wall and describes baseline functional levels in nulliparous women. Using these baseline values, we aim to compare nulliparous with postpartum women, intended to provide anatomical and functional references for decision-making prior to surgical reconstruction of the abdominal wall.

## Methods

Seventy-one healthy nulliparous female volunteers were recruited *via* social media, including Instagram and Facebook. The invitation to participate was widely spread among users, both private users as well as professional users, clinics and companies. The information was spread to different social groups and age groups in Stockholm and Sweden. Physiotherapy clinics also spread the information at their clinics in different parts of Stockholm, by putting up flyers.

The inclusion criteria were nulliparous healthy women aged 18–50 years, who had never been at any stage of pregnancy. Women who had previously undergone abdominal surgery through midline incision were excluded, but women who had undergone laparoscopic surgery or open surgery though incisions not involving the midline were considered eligible. Clinical examinations and questionnaire completion were performed at the clinic Hela Kvinnans Klinik in Stockholm. The duration of the appointment was approximately 30 min; ultrasound examination 20 min and questionnaire completion 10 min.

### Ultrasound Assessment

The General Electric ultrasound machine Logiq^®^ P9 with high resolution 50 mm linear array transducer was used. Eight ultrasound images were taken at five different locations, and in two body positions. Two exercises were filmed with the ultrasound at one location in supine lying. Three different measurements were taken with a tape measure at one location, in two different body positions. The examination was performed in both supine as well as in standing position to examine if the RD changes with body position.

All ultrasound examinations were conducted in a standard setting at the same clinic, and by one operator (KW) with 5 years’ ultrasound experience and more than five hundred hours of documented ultrasound musculoskeletal imaging (KW). The focus and depth of the ultrasound signal were adjusted to the individual depending on the anatomy, and automatic time gain control and cross beam were used. The same machine settings were used and remained as standard throughout all examinations.

### Anatomical Preferences

#### Linea Alba

**FIGURE 1:**
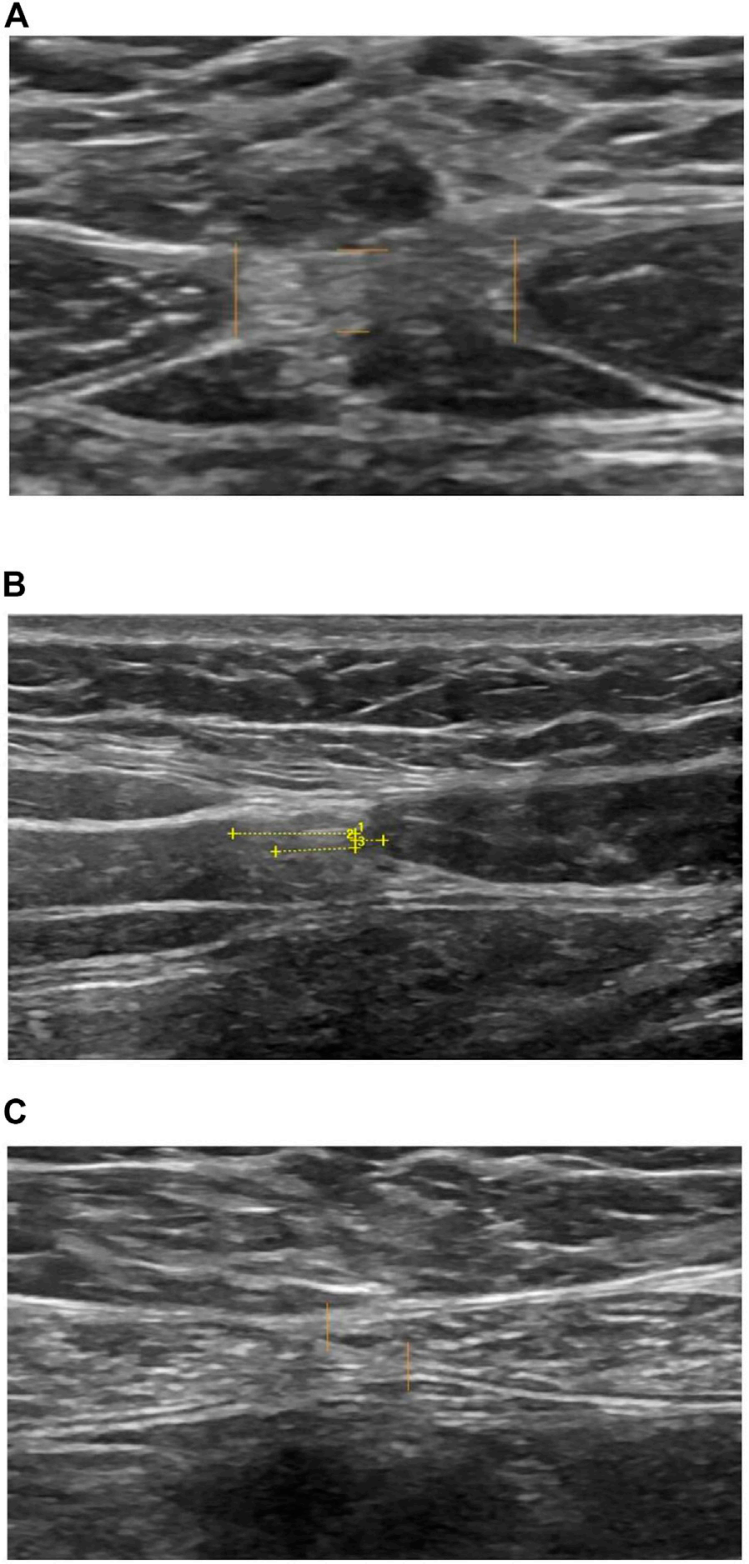
Ultrasound measurement. **(A)** Lina alba IRD and thickness. **(B)** Linea semilunaris at umbilical level. 1) Rectus abdominis muscle to external oblique muscle. 2) Rectus abdominis muscle to internal oblique muscle. 3) Rectus abdominis muscle to transverse abdominis muscle. **(C)** Linea alba 2 cm inferior to umbilicus, IRD negative value.

The separation of the medial borders of the rectus muscles were measured 3 cm superior to the umbilicus, just above the superior margin of the umbilicus, and 2 cm inferior to the umbilicus. Point zero was set at the outer margin of the umbilicus cavity. The linea alba measurements were performed at the same locations described by Beer et al. (5) and Lee et al. (13). A senior radiologist (CH) defined the most medial aspect of the muscular compartment of the rectus abdominis muscle from where measurements were performed. In cases where the muscular compartment was poorly defined at the point of measurement, we followed the innermost aspects of the posterior and anterior rectus sheaths to identify the medial confluence (Figure 1A).

#### Linea Semilunaris

There are currently no recommended locations and methods for the measurement of linea semilunaris, so we used the lateral border of the rectus muscle on the right at the level of the umbilicus and at the inferior margin of the costal arch on the right to document normal anatomical variations. The aim of this measurement was to determine the position of the lateral border of rectus abdominis in relation to the medial borders of the external oblique muscle the internal oblique muscle, and the transverse abdominis muscle where they fuse into the linea semilunaris.

Point zero was set at the most lateral aspect of the muscular compartment of rectus abdominis muscle, measuring the lateral distance to the most medial aspect of the muscular compartment of the external oblique muscle, the internal oblique muscle, and the transverse abdominis muscle respectively (Figure 1B).

**FIGURE 2:**
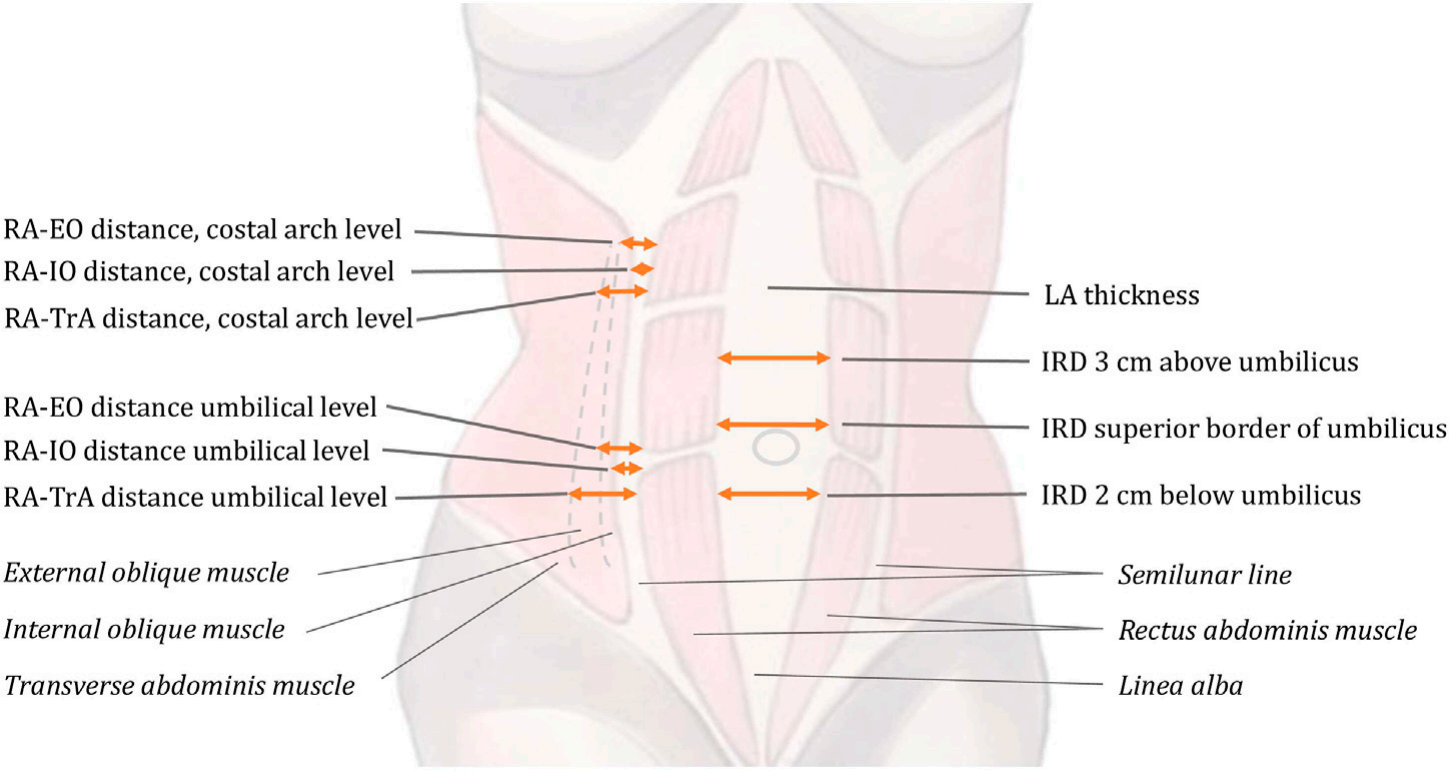
Description of the anatomical measurement points. The inter-recti distance (IRD) measured at three positions: superior border of the umbilicus; 3 cm above and 2 cm below the umbilicus. The thickness of linea alba (LA) measured above the umbilicus. Variations of the linea semilunaris anatomy are described with measurements of the distance between the lateral lining of the rectus abdominis (RA); the external oblique (EO); the internal oblique (IO); and the transverse abdominis (TA) muscles at the umbilicus level and at the coastal arch level.

In cases where the muscular compartment was poorly defined at the location of the measurement, we followed the curvatures of the innermost aspects of the posterior and anterior rectus sheaths respectively to find their lateral or medial confluence depending on the muscle examined. All standards were set in agreement with the radiologist. In one case the three examiners were not able to reach agreement as the ultrasound images did not provide the necessary information at that location. These images were excluded. The anatomical findings are described in Table 1. Measurement locations are shown in Figure 2.

**TABLE 1:** Anatomical findings with Ultrasound and tape measurement assessment. Inter-recti distance (IRD) at different levels in supine and standing positions; linea semilunaris width at umbilicus level and coastal arch level, specified on the distance between the lateral lining of the rectus abdominis (RA), the medial lining of the external oblique (EO), the internal oblique (IO) and the transverse abdominis (TrA) muscles; waist circumference tape measurements at different levels.

Ultrasound imaging supine	Mean (mm)	Range (mm)
IRD 3 cm above the umbilicus	9	4–20
IRD at the superior border of the umbilicus	10	3–24
IRD 2 cm below the umbilicus	2	−5–10
Thickness of the linea alba	3	1.5–5
Linea semilunaris width at umbilicus level		
Distance between the RA and the EO	12	−10–28
Distance between the RA and the IO	1	−14–13
Distance between the RA and the TrA	15	−14–32
Linea semilunaris width at coastal arch level		
Distance between the RA and the EO	9	−11–24
Distance between the RA and the IO	1	−9–16
Distance between the RA and the TrA	−10	−33–25
Ultrasound imaging standing		
IRD at the superior border of the umbilicus standing relaxed	12	3–26
IRD at the superior border of the umbilicus standing on one leg	13	6–37
Waist circumference tape measurements	Mean (cm)	Range (cm)
Hip bones supine relaxed	85	71–119
Hip bones standing relaxed	89	76–125
Hip bones standing on one leg	87	71–122

The ultrasound images of the linea alba and linea semilunaris were taken in the supine position with the head on a small pillow, the legs fully extended, the muscles of the abdominal wall fully relaxed during normal breathing, and the arms resting beside the body. The ultrasound film clips were taken in the supine position with the legs bent 90° at the knee joint and the spine flat with neutral lordosis.

The spontaneous curl-up was performed by asking the woman to look at her knees by raising her head and curling up the shoulders. The curl-up was interrupted when the therapist could place his hand beneath the woman with the fifth finger along the table and the first and second fingers touching the spinous process of the woman’s first thoracic vertebra. This generally occurred when the spines of the scapula were lifted from the bench. This movement was performed without further instructions. The preactivated curl-up was performed by instructing the woman to gently activate the pelvic floor and the abdominal wall muscles, breathe out, and then perform the curl-up as described above.

While standing relaxed, the woman was told to shift to a comfortable stance with the feet hip width apart, to take a deep breath, and then fully relax the abdominal wall muscles. The breathing was repeated until the woman seemed to stand fully relaxed. When balancing on one leg, the woman was asked to lift the other knee up to hip level and keep her balance. No preactivation or relaxation of the abdominal wall was done.

The anonymized ultrasound images were reviewed by the manual therapist (KW) performing all measurements, and a specialist surgeon (AO), both with more than 5 years’ experience of ultrasound imaging and interpretation. All images were also reviewed by a radiologist (CH) with more than 15 years’ experience.

#### Waist Circumference Measurements

The circumference of the abdominal wall was measured at the hip bones, at the cristae iliaca, and at the widest point of the lower abdomen (generally 2 cm below the umbilicus). The tape measure was applied directly to the skin at each location in three different positions (supine position, standing with relaxed abdominal wall, and standing on one leg) to assess any measurement difference.

In the supine position a small pillow was placed under the head, legs fully extended, abdominal muscles fully relaxed, with normal breathing and arms resting beside the body.

In standing relaxed position, the woman was instructed to stand with a comfortable stance with the feet about hip width apart, to take a deep breath, and then to fully relax the abdominal wall muscles. The breathing was repeated until fully relaxed.

While standing on one leg, the other knee was raised to hip level keeping balance. No preactivation or relaxation of the abdominal wall were done.

#### Questionnaires

To determine functional levels and link these with the results of ultrasound, participants completed questionnaires regarding urinary incontinence (UDI 6, IIQ 7) (14), low back pain (15), disability rating in everyday life (DRI) (16), habitual physical activity (Baecke) (17), and a general questionnaire of lifestyle and health (SF-36) (18).

### Statistical Analysis

Statistical analyses were carried out using SPSS 28.0. Differences between the SF-36 subscale ratings and the predicted ratings from the age-matched female background population was tested with paired t-test. The association between BMI, waist circumference and IRD were tested with Pearson’s correlation test. The sample size was determined based on assumptions regarding the variability in the population.

## Results

Mean age was 30.5 years (range 19–50 years) and mean BMI 24 kg/m^2^ (18–37). Study population characteristics are shown in Table 2. Ultrasonography showed a mean IRD of 10 mm (3–24) at the superior border of the umbilicus, 9 mm (4–20) 3 cm above the umbilicus, and 2 mm (−5–10) 2 cm below the umbilicus. Mean thickness of the linea alba was 3 mm (1.5–5). Mean distances between the lateral edge of the rectus muscle and the external oblique, internal oblique, and transverse muscles were 12 mm (−10–28), 1 mm (−14–13), and 15 mm (−14–32) respectively at the level of the umbilicus.

**TABLE 2:** Study population characteristics.

Mean age, years (standard deviation) range	30.5 (6.0) 19–50
Mean BMI (standard deviation) range	24 (3.4) 18–37
Type of living	
Living single	27 (38%)
Living with partner/engaged/married	41 (58%)
Living with parents/other adult	3 (4%)
Level of education	
Primary and high school education	11 (16%)
University education or similar	45 (63%)
Higher academic level	15 (21%)
Occupation	
Sedentary	16 (23%)
Intermediate	50 (70%)
Heavy	5 (7%)

Occupation, description of the levels. Sedentary: e.g., administrator, IT developer, accountant (Baecke work score <2); Intermediate: e.g., nurse, physiotherapist, midwife, naprapath, pharmacist (Baecke work score ≥2 to ≤4); Heavy: e.g., personal trainer, nurse w lifting, dancer (Baecke work score >4).

IRD at the superior border of the umbilicus in relaxed standing position was 12 mm (3–26) and standing on one leg 13 mm (6–37).

We did not find any statistically significant correlation between waist circumference and IRD, neither in supine position, standing relaxed or standing on one leg. There was also no correlation between BMI or any of the IRD measures. Circumference of the abdominal wall in the supine position, standing relaxed, and standing on one leg was 85 cm (71–119), 89 cm (76–125), and 87 cm (71–122) respectively.

**FIGURE 3:**
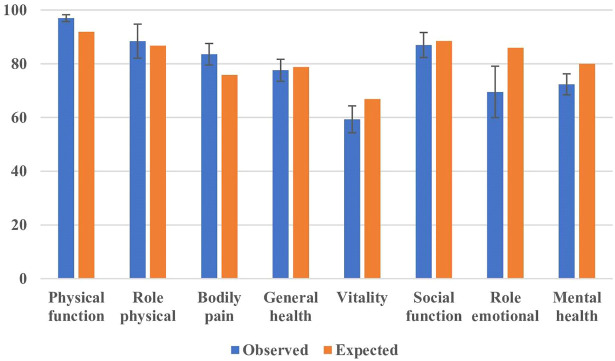
SF-36 outcome. Error bars indicate 95% confidence interval. The expected outcome was determined from an age-matched cohort of women.

Responses to the questionnaires showed generally low scores for DRI, UDI-6, IIQ-7, and Oswestry, and generally high scores for the Baecke questionnaire (Table 3). The women rated their physical function (*p* < 0.001) and bodily pain (*p* < 0.001) higher than the age-matched background population, but vitality (*p* = 0.004), role emotional (*p* = 0.001) and mental health (*p* < 0.001) lower than the background population (Figure 3).

**TABLE 3:** Questionnaires and symptom scores from the questionnaires, Baecke, oswestry, disability rating index, urinary distress inventory and the incontinency impact questionnaire.

	Mean (SD)	Range
Baecke		
Occupational physical activity	2.84 (0.80)	1.375–4.25
Sports physical activity	3.18 (1–06)	1–5.00
Leisure time physical activity	3.11 (0.64)	1.75–4.50
Oswestry (low back pain)	1.75 (3.00)	0–13
Disability Rating Index (DRI)	4.09 (6.22)	0–26.92
Urinary Distress Inventory (UDI 6)	1.80 (2.47)	0–11
Incontinence Impact Questionnaire (IIQ7)	0.28 (0.90)	0–5

## Discussion

Postpartum abdominal wall insufficiency has gained increasing attention as a condition amenable to surgical treatment or appropriate training. However, a full understanding of the impact of pregnancy on the anatomy and physiology of the abdominal wall can only be achieved when it is related to prepartum anatomy and function. The present data are derived from a cohort of seventy-one Swedish nulliparous women whose abdominal wall anatomy was examined in detail using ultrasound. The findings in this study provide several well-defined anatomical measurement points that can be used for reference values.

The level of physical activity was in general moderate to high in comparison with previous reports (19). The nulliparous women in this study rated their physical health slightly higher than the normal population, but their mental and emotional status slightly lower (Figure 3).

Most women had few or no urinary symptoms, but a small group of outliers had several symptoms, accounting for the broad range of ratings. The majority did not suffer from lower back pain or abdominal wall insufficiency. Of the participants in our study, 38 women (54%) were above the mean age of first pregnancy in Sweden.

Not only pregnancy but also age affects the anatomy and function of the abdominal wall. Some women were well over the age of 30, but none was postmenopausal. The aim of the study, however, was to investigate a broad range of fertile nulliparous women, including those who had passed the age when most women have already undergone their first delivery. This could explain why the ratings on some of the subscales of SF-36 deviated from the background population.

There are anatomical differences between individuals that might have affected the results. This is especially true regarding the location 3 cm superior to the umbilicus, where the fascia of the tendinous intersection of the rectus abdominis is located in some individuals, with a low density of muscle fibers. In these individuals it is more difficult to find the exact measurement locations for IRD as described above. In other individuals, the muscle belly of the rectus abdominis muscle is well developed at this location, and measurement is easy. This caveat applies to all studies measuring the linea alba at different locations, not only ours.

The points zero for RD measurements were set at the same anatomical points described by Beer et al. and Lee et al. (5, 13). Our findings support the new classification system suggested in the EHS guidelines from 2021 (6), where RD is defined as a separation of the rectus abdominis muscles exceeding 2 cm. In our study only one participant had an IRD exceeding 2 cm at the superior border of the umbilicus in the supine position.

We found no description of locations or methods for measurement of the linea semilunaris in the literature. We chose to locate measurements lateral to the umbilicus on the right side and inferior to the costal arch on the right side. The reason for choosing these locations is the presence of clear anatomical landmarks for reference purposes, though we are aware that the costal arch position against the spinal column can differ between individuals. We decided to measure at these locations because this provides important information when performing rectus diastasis surgery.

The advantage of ultrasonography is the possibility to measure both anatomy and muscle activity in different body positions. However, it is operator-dependent and requires adequate training. Studies have confirmed good inter-rater reliability indicating that ultrasound imaging is a reliable instrument for evaluating abdominal muscle and fascial thickness (20, 21). A complementary ultrasound examination is a feasible and valuable method that can be recommended for use in everyday clinical practice (22).

Ultrasound measurement of the abdominal wall anatomy showed lower IRD values than previous comparable findings (5) (Table 1). IRD in the supine position was 10 mm measured at the superior border of the umbilicus. When measuring the IRD 2 cm inferior to the umbilicus, we found negative values in six participants. This was because the rectus muscles overlapped at the assessed location. In 17 participants there was no separation between the rectus abdominis muscles 2 cm inferior to the umbilicus. Our findings gave a mean of 2 mm 2 cm inferior to the umbilicus, which differs from the widely accepted value of 16 mm or more when defining RD at this location.

Mean IRD when standing with the abdominal wall relaxed was 12 mm (3–26 mm) at the level of the umbilicus, which is wider than the median value in the supine position (10 mm). However, in 18 participants (25%), IRD was ≥1 mm less when standing relaxed compared to the supine position. The range of IRD in the standing position could be the result of posture, the extent to which the abdominal wall was relaxed, pain while standing, and other possibilities. Participants were not given instructions on the standing posture, only instructions on how to relax the abdominal wall. These results could serve as baseline references when comparing nulliparous women with postpartum women. No other study comparing these body positions could be found.

The mean IRD when standing balanced on one leg was 13 mm (6–37 mm) at the level of the umbilicus which is wider than mean values when supine (10 mm) and standing with relaxed abdominal wall (12 mm). However, in 14 participants (19%), IRD was ≥1 mm less when standing on one leg compared to standing relaxed. The difference in readings could be due to the lack of instruction on the standing posture. The participants were instructed to stand on one leg and lift the other knee up to hip level.

Narrowing of the IRD could possibly be caused by the combined forces of all muscle layers of the abdominal wall when standing on one leg, including the deep transverse muscle, both layers of the oblique muscles as well as the rectus abdominis muscle. The rectus abdominis muscle probably narrows the IRD. We cannot comment whether this narrowing of the IRD is the result of natural contraction together with the other deeper abdominal muscles, or if it is a result of excessive contraction of the rectus abdominis muscle itself. We did not register standing posture or compensations made to maintain balance; factors that will be investigated in a future study.

The anatomy of the semilunaris varied at both locations. A contributing factor to this could be individual variation in location of the umbilicus in relation to the costal arch. In future studies, perhaps the measurement locations should be related to levels in the vertebral column for better uniformity. Other measurement points could also be considered.

In this study we included measurement of the abdominal wall circumference with a tape measure. This is an important measurement and correlates to general health and is usually measured at the level of the hip bones (23). We used the measurement at the hip bones, usually 2 cm below the umbilical level, where the abdominal circumference usually is widest. This is due to get reference values for the nullipara group to have as baseline values to the postpartum group of women. Some participants found it difficult to fully relax the abdominal wall, especially in the standing position. Having a higher resting tone in the abdominal wall and the rectus abdominis muscles may affect the width of the linea alba (13, 23) as well as the abdominal wall circumference.

This study will be followed up by a single-blinded training study on women 3–12 months postpartum. The measurements from this observational study may provide baseline values for nulliparous women for comparison with postpartum women. They may also serve as baseline data on the anatomy of the abdominal wall in general and the linea alba specifically in nulliparous women. Although there are several possible locations to measure the linea alba, the measurement points used in this study have been used in previous studies (4, 5), are reproducible, and provide values that can be related to those derived from the other study groups.

If RD is to be considered a pathologic condition requiring surgical correction, a baseline state of the unaffected abdominal wall anatomy has to be defined. Surgery may be indicated also for symptoms presenting during the course of what may be considered a normal adaptation to pregnancies, but this may affect priorities and resource allocations. To be able to define any deviation from the normal anatomy in the postpartum period, reliable measures of the anatomy in nulliparous women are crucial.

### Limitations

Although the aim was to identify a group of women that represented the entire Swedish nulliparous population, there may have been selection bias in the recruitment process. Although it was a convenience sample, there may have been a selection of active women with expectations and demands on their functional level. Nevertheless, the questionnaire results indicate that the sample did not deviate substantially from the general population. As social media was the base for recruitment, women actively seeking information related to their own health may have been over-represented.

The age range (19–50 years) may question the external validity of the study since the mean age of first pregnancy in Sweden in 2021 was 30.1 years (24). Of all Swedish women, 13.5% were still nulliparous at the age of 45 in 2020 (25). The size of the sample was relatively small, which may limit the external validity of the study. Nevertheless, the standard intervals presented in Table 2 were relatively narrow, which indicates that a larger sample would not have substantially changed the outcome. On the other hand, a sample mixture that more accurately would have reflected the entire female nulliparous population may have provided other outcomes.

We chose waist circumference as a measure of truncal obesity as we believe this to be a more appropriate measure than weight or Body Mass Index (BMI). It is, however, possible that inclusion criteria based on BMI could have provided a cohort that would have reflected women considered for surgical management of RD in a hypothetical postpartum stage more adequately.

## Conclusion

The present data and measurement points may serve as a standard for assessing linea alba and linea semilunaris in women. More studies are needed to evaluate their clinical relevance and how they are affected by pregnancy.

## Data Availability

The raw data supporting the conclusion of this article will be made available by the authors, without undue reservation.
